# IL-2 and IL-6 cooperate to enhance the generation of influenza-specific CD8 T cells responding to live influenza virus in aged mice and humans

**DOI:** 10.18632/oncotarget.10047

**Published:** 2016-06-14

**Authors:** Xin Zhou, Jacob W. Hopkins, Chongkai Wang, Vinayak Brahmakshatriya, Susan L. Swain, George A. Kuchel, Laura Haynes, Janet E. McElhaney

**Affiliations:** ^1^ UConn Center on Aging and Department of Immunology, University of Connecticut School of Medicine, Farmington, CT, USA; ^2^ Department of Pathology, University of Massachusetts Medical School, North Worcester, MA, USA; ^3^ Health Sciences North Research Institute, Sudbury, ON, Canada

**Keywords:** aging, CD8 T cell, interleukin-2, interleukin-6, granzyme B, Gerotarget

## Abstract

An age-related decline in cytolytic activity has been described in CD8^+^ T cells and we have previously shown that the poor CD8^+^ effector T cell responses to influenza A/H3N2 challenge result from a decline in the proportion and function of these cytolytic T lymphocytes (CTL). Here, we describe that addition of exogenous cytokines to influenza-stimulated PBMC from both aged mice and humans, enhances the generation of influenza specific CD8 CTL by increasing their proliferation and survival. Our data show that the addition of IL-2 and IL-6 to splenocytes from mice previously infected with influenza virus restores the aged CD8^+^ T cell response to that observed in young mice. In humans, IL-2 plus IL-6 also reduces the proportion of apoptotic effector CD8^+^ T cells to levels resembling those of younger adults. In HLA-A2^+^ donors, MHC Class I tetramer staining showed that adding both exogenous IL-2 and IL-6 resulted in greater differentiation into influenza-specific effector CD8^+^ T cells. Since this effect of IL-2/IL-6 supplementation can be reproduced with the addition of Toll-like receptor agonists, it may be possible to exploit this mechanism and design new vaccines to improve the CD8 T cell response to influenza vaccination in older adults.

## INTRODUCTION

The current split virus influenza vaccines are designed to stimulate protective antibody responses but they often fail to provide protection in older adults. As a result, hospitalization and death rates due to influenza have continued to increase over the last two decades in spite of widespread influenza vaccination programs [1–3], providing an impetus for the discovery of more effective influenza vaccines.

A multitude of changes in the immune system occur with aging, but the specific mechanisms that increase risk for influenza illness and limit the protective effects of vaccination in older adults remain poorly understood. Age-related changes in T cell responses are associated with a decline in antibody responses to influenza vaccination [4, 5]. Nevertheless, the nature and specific mechanisms involved in declines in vaccine efficacy in old age remain unclear. We have shown that older people who develop influenza illness have low levels of the cytolytic mediator, granzyme B (GrB), and a lower ratio of IFNγ:IL-10 released from peripheral blood mononuclear cells (PBMC) following challenge with live influenza virus [6–8]. These results suggest a shift away from a more effective T helper type 1 (Th1) and related cytolytic response, toward a less effective response to influenza challenge with aging [9].

In parallel studies conducted in a mouse model of aging, we have previously demonstrated that IL-2 [10, 11] and pro-inflammatory cytokines such as IL-6 [12, 13], can restore the generation, memory and responses of CD4^+^ effector T cells from naïve CD4^+^ T cells to those seen in younger animals. Moreover, TLR-stimulation of dendritic cells (DC) can induce high IL-6 levels during cognate interaction, causing the responding T cells to produce more IL-2 [14]. In this current study, we examined the effects of IL-2 and IL-6 on the CD8^+^ T cell response to influenza virus and showed that the addition of these cytokines restored the response to influenza in aged mice to that observed in young mice.

To complement these murine experiments, we also examined age-related changes in the CD8^+^ cytolytic T cell response to influenza in human PBMC following influenza vaccination using methods previously developed in our laboratory [15]. To that end, we postulated that the addition of key cytokines *in vitro* could restore the cytolytic response of aged T cells to that seen in younger adults. Previously, we demonstrated that vaccinated older adults exhibited T cell populations with reduced proportions and numbers of memory cells [15]. In addition, the decline in naïve T cells relative to memory T cells was much more dramatic in the CD8^+^ compared to the CD4^+^ T cell compartment in older individuals. With aging, the effector T cell subset displayed diminished generation of cytolytic effector T cells, including a reduction in GrB^+^/Perforin^+^ (Perf^+^) cells and a decline in cytolytic function [15]. Furthermore, effector memory and effector CD8^+^ T-cell subsets obtained from older subjects exhibited diminished proliferative responses and cytolytic activity in response to influenza A/H3N2 challenge. These age-related declines in proliferative responses and cytolytic activity were much less marked in the corresponding CD4^+^ as compared to CD8^+^ T cell subsets [15]. We postulated that these results could be attributed to changes involving the CD8^+^ T cell subset, as these are driven to a late stage or terminally differentiated state where they lose cytolytic function [16, 17]. Consistent with this hypothesis, GrB continues to be expressed in a large proportion of these CD8^+^ T cells but in the absence of Perf [7, 15, 18] and thus cannot contribute to cytolytic activity against influenza virus-infected cells.

In a pre-clinical model using human PBMC to test different adjuvants combined with split-virus influenza vaccines (SVV), we have shown that addition of toll-like receptor (TLR) agonists can be used to improve the IFNγ:IL-10 ratios as well as GrB responses to influenza challenge [19]. The addition of a TLR4 agonist, Glucopyranosyl Lipid Adjuvant-Stable Emulsion (GLA-SE), stimulated myeloid dendritic cells to produce inflammatory cytokines (i.e., TNFα, IL-1, IL-6). This effect was associated with a dramatic reduction in IL-10 levels in response to influenza challenge when PBMC were pre-treated with TLR4/SVV compared to SVV alone [19]. The experiments reported herein analyze the mechanism for these observations.

With the above considerations in mind, we sought to explore the hypothesis that enhanced levels of key cytokines would improve the response of aged human T cells to influenza virus challenge. As part of this effort, we tested recombinant IL-2, IL-6, IL-4, IL-10 and IL-17A, selected on the basis of existing cytokine assay data, in order to evaluate the capacity of other potential key regulatory cytokines to reverse age-related declines in CD8^+^ T cells. We observed that PBMCs from older adults produce lower IL-2 levels and higher IL-6 levels following an influenza challenge when compared to those from younger individuals. Nevertheless, supplementation with a combination of IL-2 and IL-6 was most effective in reversing age-related defects in CD8^+^ T cell responses to influenza, thus offering important evidence supporting the clinical potential of selecting more effective adjuvants as part of an effort designed to improve the effectiveness of influenza vaccines in older people.

## RESULTS

### Granzyme B expression by murine memory CD8+ T cells can be enhanced by the addition of IL-2 and IL-6

**Figure 1:**
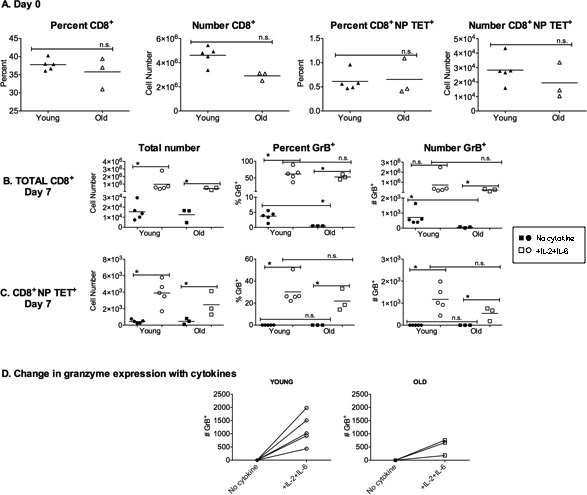
NP-specific memory CD8^**+**^ T cells *in vitro* response to influenza can be augmented by the addition of cytokines Mice were infected 4 weeks prior with 500 EID_50_ PR8 (H1N1) influenza to generate a lasting immune response. **A.** Splenocytes were analyzed on the day of harvest (day 0) by flow cytometric analysis of cells from individual young (4 month) and aged (18 month) mice. Graphs show the percent and total number of CD8^+^ and NP-specific CD8^+^ T cells (NP TET+). **B.**-**D.** Splenocytes from individual mice were then cultured for 7 days with 50 EID_50_ PR8 (H1N1) with or without IL-2 and IL-6 (10ng/ml). **B.** Analysis of total CD8^+^ T cells after 7 days of culture for percentage and total numbers of CD8+ T cells expressing granzyme B (GrB). **C.** Analysis of total NP tetramer-specific CD8+ T cells for percent and total numbers of cells with GrB expression. **D.** Comparison of total number of GrB expressing cells after 7-day secondary response to PR8 virus with and without IL-2 and IL-6. Each symbol represents a single animal. *p < 0.05. n.s. = not significant.

GrB is an important effector molecule used in fighting viral infections and declines in expression could negatively impact viral clearance. In order to examine how aging affects the memory CD8^+^ T cell response to influenza infection and GrB expression, young and old mice were infected with a sublethal dose of influenza A/PR/8/34 (PR8), and splenocytes were analyzed one month later. Figure 1A shows that the percent and total number of CD8^+^ T cells in the spleens of these mice were not significantly different. In addition, the percent and number of NP-specific CD8^+^ T cells in the spleen was not significantly different between young and old groups. These splenocytes were then cultured for 7 days with influenza virus with or without added IL-2 and IL-6. These two cytokines were chosen based on their ability to enhance the function of memory CD4^+^ T cells from old mice [11]. On day 7, total CD8^+^ T cells were analyzed (Figure 1B). In both young and old groups, the addition of IL-2 and IL-6 to splenocyte cultures increased the total number and the percent of CD8^+^ T cells recovered and total number of GrB expressing CD8^+^ T cells. Importantly, the young group stimulated with virus alone expressed a higher percentage and number of GrB expressing cells compared to the old group, but this difference was overcome by the addition of the cytokines.

A similar pattern was also observed for influenza-specific CD8^+^ T cells. Influenza nucleoprotein (NP)-specific CD8^+^ T cells were detected with a MHC Class I tetramer. In both young and old groups, the number of NP-specific CD8^+^ T cells was increased in cultures containing added IL-2 and IL-6 (Figure 1C). While there were almost no GrB expressing NP-tetramer^+^ cells in both young and old groups stimulated with virus alone, the addition of IL-2 and IL-6 significantly enhanced GrB expression, regardless of age. This increase in GrB expression in NP-tetramer^+^ cells is also shown in Figure 1D for each individual young and old animal. Thus, overall, the addition of IL-2 and IL-6 to murine memory CD8^+^ T cells can boost their GrB expression, which could aid in viral clearance during an infection. Our studies then went on to determine if a similar enhancement could also be observed in human CD8^+^ T cells.

### Older human CD8^+^ effector memory T cells exhibit increased apoptosis and diminished proliferative capacity in response to influenza when compared to young

**Figure 2:**
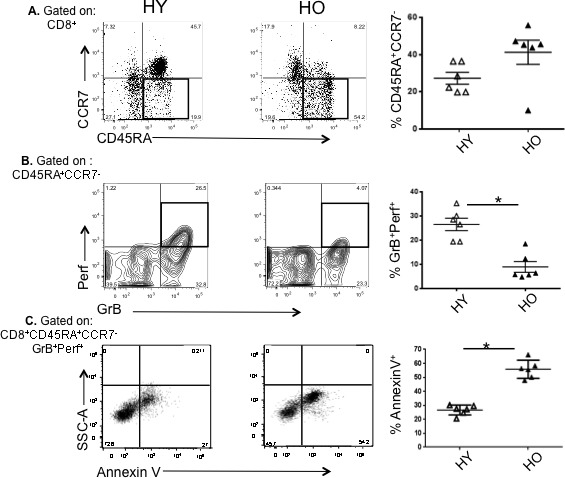
High apoptotic tendency and decline in proliferative capacity in effector memory CD8^**+**^ T cells responding to influenza virus in older compared to young controls Human peripheral blood mononuclear cells (*n* = 6/group) obtained 10 or 20 weeks post-vaccination were cultured for 20 hr in the presence of influenza A/H3N2 live virus. **A.** Graphs show a representative scatter plot and compiled results of the proportion of CD8^+^ T cells that are CD45RA^+^CCR7^−^ following influenza challenge. **B.** Gating on CD8^+^CD45RA^+^CCR7^−^ T cells, the proportion of cytolytic effectors in the boxes (GrB^+^Perf^+^) in the representative scatter plots and graphs of the compiled results are shown. **C.** Gating on CD8^+^CD45RA^+^CCR7^−^GrB^+^Perf^+^ T cells, the proportion undergoing apoptosis (Annexin V^+^) are shown in the representative scatter plots and graphs of compiled results. Error bars represent standard error of the mean. *p < 0.0001.

We have previously shown that CD8^+^ effector T-cell subsets isolated from older adults, which co-express intracellular GrB with Perf (GrB^+^Perf^+^) or exhibit the CD127^+^CD25^±^ phenotype, also demonstrate strong cytolytic activity in response to virus challenge at 4 weeks following influenza vaccination. However, the response tends to be short-lived, with a significant reduction in the frequency and influenza-specific cytolytic activity involving CD8^+^ T cells by 10 weeks post-vaccination [15]. Therefore, we now compare responses involving CD8^+^ T cells obtained from young and older individuals 10-20 weeks post-vaccination and stimulated *ex vivo* with influenza. Most T cells found in PBMC at this time express an effector memory phenotype [15]. PBMC were isolated and stimulated with live influenza virus. After 20 hours in culture, older compared to young adults showed a higher proportion of total effector memory CD8^+^ (CD45RA^+^CCR7^−^) T cells (Figure 2A) but a significant reduction in the subset expressing GrB and Perf effector molecules following virus challenge (Figure 2B). This indicates that the generation of memory cells is not diminished with aging, but that their functional cytolytic capacity is likely impaired. We next analyzed this CD45RA^+^CCR7^−^GrB^+^Perf^+^CD8^+^ T cell subset, which represent the putative memory cytotoxic population, by Annexin V staining as an indicator of apoptosis. Approximately twice as many of the GrB^+^Perf^+^ effector memory CD8^+^ T cells from older subjects expressed Annexin V, indicating that more than half of this subset was undergoing an early stage of apoptosis (Figure 2C). This suggests that with aging the memory CD8^+^ T cell population that has CTL potential becomes more prone to apoptosis, which is responsible in some part for the generation of fewer cytotoxic effectors.

### IL-2 and IL-6 enhance the proliferative capacity of human older CD8^+^ T cells and its memory effector subsets in response to influenza virus

**Figure 3:**
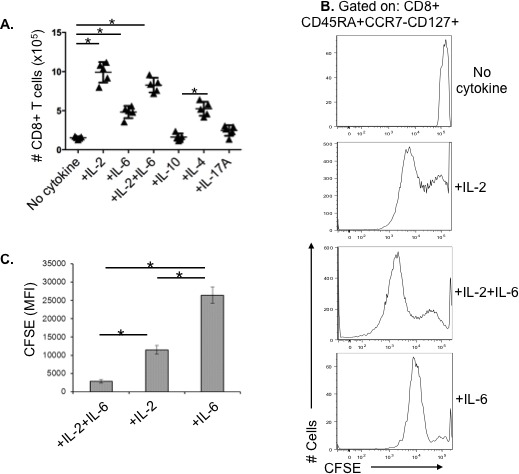
The restorative effect of recombinant cytokines on aged CD8^**+**^ T cells and the proliferative capacity of its effector memory subset in older adults **A.** Human CD8^+^ T cells (*n* = 5/group) from healthy older adults (70-80 years old) were sorted and stimulated with influenza H3N2 virus for 7 days with or without cytokines, then total CD8^+^ T cell numbers were counted. **B.** Representative histogram of human CD8^+^ T cells from a healthy older adult were sorted and stimulated with influenza H3N2 live virus for 3 days in the presence or absence of recombinant IL-2 (10 ng/mL) and/or IL-6 (15 ng/mL), cells were stained with CFSE at a concentration of 1μM, and then cultured in fresh medium containing cytokines for another 3 days before FACS analysis. **C.** Compiled data (*n* = 5/group) showing the mean fluorescence intensity (MFI) of the CFSE staining demonstrating that the highest level of proliferation (lowest MFI) occurs with the addition of both IL-2 and IL-6. Error bars represent standard error of the mean. *p < 0.0001.

It has been shown that the addition of exogenous IL-2 can maintain effector T cell proliferation, block apoptosis, and when added to *in vitro* cultures, can largely overcome the age-related decline in naive CD4^+^ T-cell expansion [10, 11]. We have also found in murine studies that IL-6 plays a key role in enhancing expansion and survival of older adult naive CD4^+^ T-cells [10, 14]. Based on our observation of a major decline in the production of IL-2 in aged human PBMC in response to influenza challenge (unpublished data) and *in vitro* studies in the mouse, we designed experiments to evaluate the effect of possible key cytokines including IL-2, IL-6, IL-4, IL-10, and IL-17A, on the generation of cytotoxic effector CD8^+^ T cells in response to influenza virus. To eliminate the effects of other PBMC on the observed responses, enhance influenza virus antigen presentation, and target T cell proliferation and differentiation, we sorted CD14^+^ monocytes, and CD4^+^ and CD8^+^ T cells from older individuals and cultured them at a 2:2:1 ratio with live influenza virus for 7 days with or without the addition of IL-2, IL-4, IL-6, IL-10, or IL-17A. Only IL-2, IL-4, and IL-6 showed a significant effect on the number of aged CD8^+^ T cells, with IL-2 having the greatest effect on enhancing cell number on day 7 of culture (Figure 3A); IL-4 was a weaker stimulus than IL-2 in terms total cell numbers, with no impact on the tendency of aged effector memory CD8^+^ T cells to undergo apoptosis when compared to control cultures with no cytokines added (data not shown). Similar effects of cytokine supplementation were observed in young CD8^+^ T cells (Supplemental Figure 1). The effect of IL-2 and IL-6 on proliferation of CD8^+^ memory T cells from older adults was also assessed by CFSE staining. Figure 3B shows representative histograms of CFSE staining and compiled results of Mean Fluorescence Intensity (MFI) are shown in Figure 3C. Addition of both IL-2 and IL-6 together during culture with influenza virus induces significantly enhanced proliferation of these CD8^+^ T cells from older subjects.

### IL-2 and IL-6 enhance CD8^+^ effector memory T-cell cytotoxicity both by blocking apoptosis and increasing expansion

**Figure 4:**
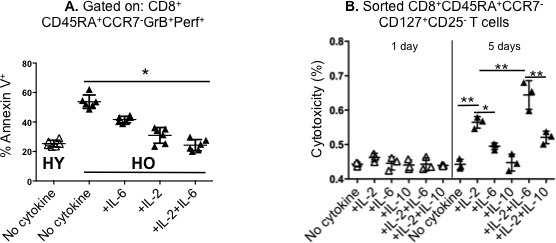
The restorative effect of different combinations of recombinant IL-2 and/or IL-6 on the apoptotic tendency and cytotoxicity in older CD8^**+**^ effector memory T cells Human peripheral blood mononuclear cells (PBMCs) from healthy young (HY) and older (HO) adults were stimulated with influenza H3N2 virus with or without cytokines (recombinant IL-2 [10 ng/ml], IL-6 [15 ng/ml], IL-10 [10 ng/ml]). **A.** Gating on CD8^+^CD45RA^+^CCR7^−^GrB^+^Perf^+^ T cells after 20 hours in culture, Annexin V staining shows that the apoptotic tendency of the HO cytolytic T cells is diminished by the addition of IL-2 and IL-6; the combined effect of these two cytokines in older adults reduces the frequency of apoptotic cells to that found in HY (*n* = 6/group). **B.** For the cytotoxicity assays, HO PBMCs (*n* = 3/group) were stimulated with virus for 1 day (open triangles) or 5 days (filled triangles) in medium with or without cytokines and the cytolytic subset (CD8^+^CD45RA^+^CCR7^−^ CD127^+^CD25^−^ T-cells) was sorted for the LDH release assay as described in Methods. The highest level of cytotoxicity was achieved with the addition of IL-2 and IL-6. Error bars represent standard error of the mean. *p < 0.0001, **p < 0.005.

Given the ability of IL-2 and IL-6 to enhance proliferation of effector memory CD8^+^ T-cells in samples from older individuals, we investigated whether IL-2 and IL-6 could also improve CTL function in the older adult PBMC cultures by additional mechanisms. First, we tested the effects on the apoptosis of CD8^+^ effector memory T cells with a cytolytic phenotype, GrB^+^Perf^+^. We found that the addition IL-2 plus IL-6, resulted in a significant decline in the proportion of Annexin V^+^ cytolytic effector T cells (CD8^+^CD45RA^+^CCR7^−^GrB^+^Perf^+^ T cells) that were undergoing apoptosis (Figure 4A). IL-2 or IL-2 plus IL-6 had a greater effect than IL-6 alone, which was not statistically different from no added cytokines in this assay. Added IL-2 plus IL-6 or IL-2 alone significantly decreased the proportion of apoptotic cytolytic cells in older adult CD8+ T cells, such that the proportion of apoptotic cells observed was similar to that found in the young adult response in the absence of any added cytokines. Thus, the response of the apoptotic older effector memory CD8^+^ T cells was dramatically improved by the addition of IL2 plus IL-6 so that it became similar to that of young cells.

Next, we evaluated the effect of IL-2 and IL-6 on the cytolytic activity of CD8+ memory effector T cells. The addition of IL-2 or IL-6 to cultures of CD8^+^ T cells from older individuals significantly enhanced the cytolytic activity of 5-day effector CD8+CD45RA+CCR7- T-cells against influenza-infected targets, and the addition of IL-2 plus IL-6 showed significantly greater cytolytic function than either IL-2 or IL-6 alone, suggesting the presence of potentially interacting effects (Figure 4B). At the same time, IL-10, used as a control, had no effect on cytolytic function (Figure 4B). No changes in cytolytic activity were observed after only one day of culture. Thus IL-2, especially in combination with IL-6, enhanced proliferation, reduced death and enhanced the cytolytic activity generated from effector memory CD8^+^ T cells.

### The decline of influenza-specific older CD8^+^ T cells from HLA-A2 donors can be recovered by adding IL-2 plus IL-6

**Figure 5:**
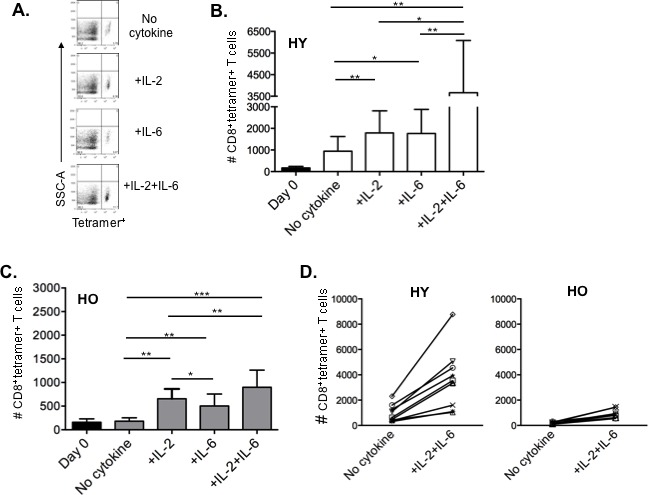
IL-2 and IL-6 induced proliferation of influenza-specific CD8^**+**^ T cells in HLA-A2^**+**^ donors Human PBMCs from healthy young (*n* = 8) and healthy older (*n* = 9) HLA-A2+ donors were stimulated with influenza H3N2 virus with or without cytokines (IL-2 [10 ng/ml], IL-6 [15 ng/ml) for 7 days, then stained with CD3, CD8, and MHC Class I tetramer to identify influenza-specific T cells. **A.** Representative dot plots gated on CD3^+^CD8^+^ T cells showing the proportion of MHC Class I tetramer^+^ cells for each culture condition. **B.**, **C.** Comparison of IL-2 and/or IL-6 induced enhancement of influenza-specific CD8^+^ T cells on Day 0 (black bars) and Day 7 in young (B, white bars) and older (C, gray bars) adults. Error bars represent standard error of the mean. *p < 0.0001, **p < 0.01, ***p < 0.05. **D.** Graphs showing an approximate 4-fold increase in MHC Class I tetramer^+^ CD8^+^ T cells in both young **D.** and older **E.** adults when cultured with the addition of IL-2 plus IL-6 compared to no cytokines. Each symbol represents a single subject from graphs **B.** and **C.**.

In order to track the *in vitro* response of influenza-specific CD8^+^ T cells from our young and old cohorts, we identified them with a MHC Class I tetramer containing the immunodominant influenza M1 epitope (GILGFVFTL). Figure 5A shows the flow cytometric analysis of tetramer-positive CD8^+^ T cells from one subject and demonstrates that addition of IL-2 plus IL-6 enhances the percentage of tetramer positive cells. Results compiled from young (Figure 5B) and older (Figure 5C) subjects also show this effect of IL-2 plus IL-6 on influenza-specific cells after 5 days of culture. Importantly, although the young adults have higher frequencies of M1 tetramer^+^ cells, there is approximately a 4 to 5-fold increase in the frequency of these cells in both young and older adults in response to influenza challenge with the addition of IL-2 plus IL-6 to the cultures. The change in *in vitro* expansion of tetramer-specific CD8^+^ T cells with the addition of IL-2 plus IL-6 when compared to culturing with the virus alone is also shown for individual young and older subjects (Figure 5D).

## DISCUSSION

The response of PBMC from older individuals to live influenza virus *in vitro* is significantly reduced when compared to the response from younger individuals, with a lower frequency of cells responding and with reduced cytotoxic activity. Here we report that addition of exogenous IL-2 and IL-6 to influenza challenged PBMC, enhances the expansion and survival of the aged CD8^+^ T cell recall response, leading to more effector CD8^+^ T cells with higher cytotoxic activity. It should be noted that our human PBMC model system includes sorted monocytes and CD4^+^ and CD8^+^ T cells so that results are not confounded by individual subject differences in the numbers of cells within these different cell subsets in the PBMC culture, while at the same time supporting the critical interactions between these subsets in the generation of the responses measured.

Studies *in vitro* with murine naive CD4^+^ T cells have previously shown that TLR agonists acting on dendritic cells (DC) cause enhanced production of IL-6 that with other elements dramatically enhances the responses of old naïve CD4^+^ T cells interacting in a cognate fashion with those DC [14, 20]. The CD4^+^ T cells divide more, undergo less apoptosis and produce more IL-2 [14]. In addition, a cocktail of pro-inflammatory cytokines, IL-6, plus IL-1 and TNFα added to cultures or to mice containing old naïve CD4^+^ T cells, results in striking enhancement and importantly in increased help for B cells [12, 13]. Thus, IL-6 in addition to an early described role as a B cell growth factors, is well-established as a mediator of CD4 and CD8 T cells function and is important for keeping T cells alive as they respond [21, 22]. In the model used in the study presented here, responses are assessed following *ex vivo* influenza virus challenge of PBMC from recently immunized donors, so it is likely that the responding CD8^+^ T cells are recently generated memory T cells. Thus these results suggest that in older individuals, the population of influenza-specific memory T cells generated by vaccination, or re-called from earlier exposures, are defective, and that the combination of IL-2 and IL-6 is able to act together to substantially enhance responses. The fact that following vaccination, IL-2 plus IL-6 can improve human CD8^+^ T cell responses that are impaired in older adults, suggests that this pathway for enhanced response is present in both mice and humans and that it can act both on the memory CD8^+^ cells generated by vaccination, as well as on naïve CD4^+^ T cells. We note that aged T cell responses are enhanced by IL-2 and IL-6 (shown herein), and by TLR agonist activated DC in both young and old mice [14, 20]. Similarly, we have previously shown that pro-inflammatory cytokines enhance proliferation and IL-2 production by aged naive CD4^+^ T cells in an NFκB-dependent manner [12, 13]; we have shown this both *in vitro* and *in vivo* with the pro-inflammatory cytokines used as an adjuvant. We suggest that IL-6 acts as a “third” signal during TCR stimulation of CD4^+^ T cells and provides co-stimulation signals leading to enhanced responses. Thus it seems aged T cells retain some of their ability to respond effectively under conditions that induce highly activated DC and pro-inflammatory cytokines, such as those that occur with live replicating pathogens, but that their response to less “dangerous” challenges, such as isolated foreign proteins or inactivated vaccines is impaired. One can speculate that this shift may to some extent actually be advantageous since it could limit potential for the development of over-exuberant responses to non-pathogens or self antigens.

It has been proposed that enhanced inflammatory responses to viral infections could be protective in terms of host defense, while at the same time “inflammaging”, overproduction of inflammatory molecules such as IL-6 with aging, has been linked to an increased risk of age-related frailty, morbidity and disability. In contrast, anti-inflammatory responses involving IL-10 and other mediators could potentially weaken resistance to infection, while contributing to healthy aging [23]. In determining the relevance of the above paradigm to our findings, we found that among the cytokines measured in our panel, the release of IL-1β, IL-6, MIP-1α and GM-CSF in *ex vivo* influenza-challenged PBMC was significantly increased in older compared to young adults. We observed that while production of both IFNγ and IL-10 in whole PBMCs declined with age, the IFNγ:IL-10 ratio declined, suggesting a possible shift toward a more anti-inflammatory response to influenza. Our previous experiments showed that this decline is independent of age cohort and history of childhood exposure to different subtypes of influenza A [9], suggesting that the dysregulation of cytokine production is an intrinsic alteration in immune function that could be targeted in the development of immunotherapies including vaccines. One way to reconcile these divergent considerations is to suggest that the constitutive systemic production of pro-inflammatory cytokines may desensitize T cell responses and lead to other negative consequences of inflammation, while increases in targeted pro-inflammatory cytokines produced by antigen-presenting cells in response to infection, that interact with responding T cells, may strikingly enhance the response of those cells (Reviewed in [24]).

It has been previously shown that both dendritic cell (DC) and CD28 costimulation are required for maintenance of the effector phase of influenza-specific CD8^+^ T cell responses *in vivo* [25]. IL-2 treatment has been shown in the mouse to restore the effector phase of the response to influenza in the absence of CD28 costimulation [26]. In humans, the loss of CD28 expression on CD8^+^ T cells in association with the late stage or terminal differentiation of these memory T cells would predict a defective effector phase and early apoptosis of the effector CD8^+^ T cells responding to influenza challenge. Our results confirm this prediction showing that reduced cytolytic activity in older compared to young adults is associated with higher frequencies of effector CD8^+^ T cells undergoing apoptosis and a decline in the proliferative response to the virus. Furthermore, we show that IL-2 supplementation could reduce the rate of apoptosis in effector CD8^+^ T cells and improve the proliferative response to the virus, and when combined with IL-6, may restore these responses to those demonstrated in young adults. While the increase in effector CD8^+^ T cells expressing the IL-2 receptor (CD25) was associated with a decline in the proportion of CD25^+^ T cells with a naïve phenotype, it is likely that this naïve subset represents the previously identified subset of CD8^+^CD45R0^+^CD25^+^ T cells which efficiently expand and differentiate into functional effector T cells upon antigenic challenge [27].

Our previous studies have documented a defect in the frequency and cytolytic activity of CD8^+^ T cells responding to a live influenza virus challenge following vaccination in older compared to young adults. Further, the duration of the response to influenza vaccination is more short-lived in older adults. In this study, we showed that this decline in the frequency and cytolytic function of effector CD8+ T cells (GrB^+^Perf^+^) is associated with increased apoptosis in the responding cells in older compared to young adults. Proliferation assays confirmed that this was associated with a loss of effector CD8^+^ T cells in the older adult PBMC. An earlier report found a similar increase in apoptosis during the response of naïve CD4^+^ T cells from old mice [14, 28], suggesting a shift in susceptibility to cell death during response may be a general characteristic of aged T cells. The sharing of this set of pathways for enhancing T cell responses between mice and humans, between CD4^+^ and CD8^+^ T cells and between naïve cells studied in mice and memory cells studied here suggests this is a well-conserved and functionally important, general pathway in T cells.

While it has been previously shown that both IL-2 and IL-6 can reverse age-related changes in T cell proliferation and function [10, 13, 29], we found that the combined effect of these two cytokines improved the proliferative response to live influenza challenge, with an associated reduction in the proportion of old effector T cells undergoing apoptosis. The addition of IL-6 resulted in a small increase in the differentiation of effector memory T cells relative to the effect of IL-2 but there appeared to be interacting effects between the two cytokines that resulted in most of the effector memory CD8+ T cells becoming effector T cells over the 5-7 days of culture.

We have previously shown that a TLR4 agonist, GLA, when added to a split influenza virus vaccine, improved the CD8^+^ T cell response of older individuals [19] in association with enhanced IL-6 production by myeloid DC in the cultures. Our previous results with this TLR4 agonist would suggest that adjuvants could be added to influenza proteins or split-virus vaccines to stimulate key cytokines including IL-2 and IL-6 and activate cytokine signal transduction pathways to enhance the proliferation and differentiation potential of memory and effector T cells in older adults.

In conclusion, the ability of IL-2 and IL-6 supplementation to restore the response of influenza-specific memory CD8^+^ T cells to become functional effectors in older adults, to levels close to those observed in young adults in response to influenza challenge, provides a mechanism that could be targeted in the design of new influenza vaccines. Most importantly, these results raise the possibility of adding adjuvants to currently approved influenza vaccines to improve vaccine efficacy in the older population most vulnerable to the serious complications of influenza.

## MATERIALS AND METHODS

### Ethics statement

This study was performed under strict accordance with the recommendations from the Guide for the Care and Use of Laboratory Animals. All animals were cared for according to local, state, and federal regulation in accordance with protocol approved by the University of Connecticut Health Center Institutional Animal Care and Use Committee (A3471) and consistent with National Institutes of Health guidelines for the care and use of animals.

All human subjects were recruited following written informed consent. The Institutional Review Board of the University of Connecticut Health Center approved all protocol and informed consent documents.

### Mice

Young (4 months) C57BL/6J male mice were purchased from the Jackson Laboratory and aged (18 months) C57BL/6 male mice were obtained from the National Institute on Aging colony.

### Virus

Mice were anesthetized with Isofluorane (4% 2.0L/min) and infected with A/PR/8/34 (PR8), intranasally at 500 EID_50_ in 50 uL PBS. Mice were monitored daily for weight loss for 21 days and removed from the study if weight loss exceeded 30%.

### Culturing murine splenocytes

Single cell suspensions of splenocytes were generated by passing individual spleens through a 70 um filter and lysing red blood cells with 1.0ml ACK (Gibco) for 1 minute. Splenocytes were cultured at 10^6^ cells/ml and stimulated with 50EID_50_ PR8 influenza for 7 days. Half of these cultures were supplemented with 10ng/ml interleukin 2 and 10ng/ml interleukin 6 (R & D Systems).

### Flow cytometry of murine splenocytes

Freshly harvested splenocytes and cells cultured for 7 days were stained with anti-CD3ε PerCP/Cy5.5 (Clone: 145-2C11, BioLegend), anti-CD-8 PE-Cy7 (Clone: 53-6.7, eBioscience) and anti-GranzymeB AlexaFluor700 (Clone: GB11, BD Biosciences). A MHC class I peptide tetramer specific for influenza virus NP_366-374_/D^b^ conjugated to PE was obtained from the Molecular Biology Core Facility at Trudeau Institute (Saranac Lake, NY). Splenocytes and day 7 effectors were incubated on ice for 60 minutes with the Class I tetramer prior to staining with surface antigens on ice for 30 minutes. Cells were permeabilized according to the BD Cytofix/Cytoperm protocol and subsequently stained for intracellular proteins. Data were acquired on the BD LSR II flow cytometer and analyzed using FlowJo 9.3.3 software.

### Human subjects

Twenty-four healthy young (20-30 years old) and twenty-three older adults (≥65 years old) from the Greater Hartford Area, CT, USA, were studied at 10-weeks or 20-weeks post-vaccination in the winter of 2011-2012 and 2014-2015. For both age groups, subjects were excluded for any acute respiratory illness presenting in the 2 weeks prior to study enrolment, insulin-requiring diabetes, advanced heart failure or lung disease, any condition or medication causing immunosuppression (i.e., prednisone > 10mg/day), or any contraindications to influenza vaccination.

### Cell culture and virus stimulation

Human PBMC were isolated from venous blood samples by Histopaque gradient purification and stimulated in 0.5 ml of AIM V media (Gibco) containing 1.0×10^6^ PBMC and 4×10^6^ TCID_50_ of influenza A/Victoria/3/75 (H3N2) virus for 20 hours as previously described [15]. For cytotoxicity assays, cells were activated with Dynabeads Human T-Activator CD3/CD28 (Invitrogen) for 6 hr, the Dynabeads removed, and then the cells were stimulated with live influenza virus for 5 days in AIM V medium containing 10% Human AB serum (Sigma). For cell surface and MHC-Class I tetramer staining, CD14^+^ monocytes and CD4^+^ and CD8^+^ T cells were sorted and mixed at a 2:2:1 ratio and stimulated with live influenza virus for 7 days before staining with tetramers and antibodies to other T cell surface markers. Recombinant cytokines were added at concentrations of 10 ng/ml for IL-2, IL-4, IL-10 and IL-17A, and at 15 ng/ml for IL-6 (R&D).

### Flow cytometry and antibodies

To determine the phenotype of T cells responding to influenza challenge, PBMCs were prepared and stained as previously described [15]. Briefly, the cells were washed with cold 0.2% FBS/PBS twice before adding the following conjugated monoclonal antibodies: Human anti-CD8-FITC (OKT8), anti-CD25-APC-Cy7 (eBioscience), anti-CD127-Biotin/PE-Texas Red, anti-CCR7 PE-Cy7 (3D12) and anti-CD8-APC-Cy7 (SK1) (BD Bioscience), anti-CD45RA-eFluor 450 (HI100) and anti-CD4-Alexa Fluor 700 (OKT4) (eBioscience). For intracellular staining, cells were fixed with 2% paraformaldehyde and permeabilized with permeabilization buffer (eBioscience), then incubated with anti-GrB Alex Fluor 647 (GB11) and anti-Perforin PE (δG9), or human anti-IL-2-FITC (MQ1-17H12), anti-IL-4-PE (MP4-25D2), anti-IL-6-V450 (MQ2-13A5), anti-TNF-α-PE-Cy7 (MAb11) (BD Bioscience) anti-IFN-γ-PerCP-Cy5.5 (4S.B3) (eBioscience) or anti-Stat3 (pS727)-Alexa Fluor 488 (49/p-Stat3) and anti-Stat5 (pY694)-Alexa Fluor 647 (47/Stat5) (BD Bioscience) antibodies. For intracellular cytokine staining, Brefeldin A solution (eBioscience) was added 5 hours before staining. For Annexin V staining, cells were cultured with serum free medium for 4 hours, then washed with binding buffer and incubated with fluorochrome-conjugated Annexin V (eBioscience). For CFSE staining, CD8^+^ T cells were stimulated with virus for 3 days with or without cytokines, CFSE (eBioscience) was added into cell culture at the concentration of 1 μM for 10 min at room temperature, and then the cells were washed and cultured in fresh medium for another 3 days. For influenza-specific MHC Class I Tetramer Staining in HLA-A2 Donors, human PBMC were prepared and stimulated with influenza A/Victoria/3/75 (H3N2) virus for 7 days in 0.2 ml of AIM V media containing 5×10^5^ PBMC in a 96 well flat-bottom plate. The MHC Class I human Tetramer-Streptavidin-Allophycocyanin (iTAg^TM^ MHC Class I human Tetramer-SA-APC, MBL International Corporation, Woburn, MA) which is used for HLA-A2^+^ donors and is specific for influenza M1 (GILGFVFTL). Cells were stained with surface markers and tetramer for one hour at room temperature. For cell sorting, human PBMC were prepared as above and sorted by using FACS Vantage SE/DIVA High Speed Sorter (BD Biosciences) running DIVA 5.0 Software. Data were acquired on the BD LSR II flow cytometer and analyzed using FlowJo 9.3.3 software.

### Cytotoxicity assay

Human PBMCs were prepared and cultured in AIM V medium containing 10% human AB serum (Sigma) with influenza A/Victoria/3/75 stimulation for 5-6 days. CD8^+^ T cells were sorted into different effector subsets defined by antibody staining before they were added to the cytotoxicity assay. Anti-CD3/CD28 activated autologous lymphoblast target cells were infected with influenza H3N2 virus for 30 min and added into V-bottom 96-well plate at a dilution of 10^4^ cells/100 μl per well in triplicate before adding sorted effector T cell subsets at E/T (effector/target) ratios of 5:1 and 3:1; co-cultures were incubated for 4hr at 37°C [15]. Lactate dehydrogenase (LDH) activity released in the cell free supernatants was determined using the CytoTox 96^®^ Non-Radioactive Cytotoxicity Assay (Promega).

### Statistical analysis

Unpaired *t-*tests were used to compare the two age groups for cytokine levels, GrB activity, and frequencies in the different CD8^+^ T-cell subsets. Analysis within age groups was done by the paired Student's t test. Analysis of variance (ANOVA) was used for comparisons across different culture conditions and adjusted using the Tukey HSD for multiple comparisons. Non-parametric tests were used where the data was not normally distributed. Analyses were performed using Origin Pro 8.5 (OriginLab, MA) and SPSS 16.0 (Chicago, IL). *P* values < 0.05 were considered statistically significant.

## SUPPLEMENTARY MATERIAL FIGURE


