# Supplementary material for: IL-2 and IL-6 cooperate to enhance the generation of influenza-specific CD8 T cells responding to live influenza virus in aged mice and humans
Source: Oncotarget. 2016 Jun 14;7(26):39171–83. doi: 10.18632/oncotarget.10047 (PMC5129923; doi:10.18632/oncotarget.10047)
Supplement: Supplementary file 1 [file oncotarget-07-39171-s001.pdf]

IL-2 and IL-6 cooperate to enhance the generation of influenza-specific CD8 T cells responding to live influenza virus in aged mice and humans

Supplementary Material

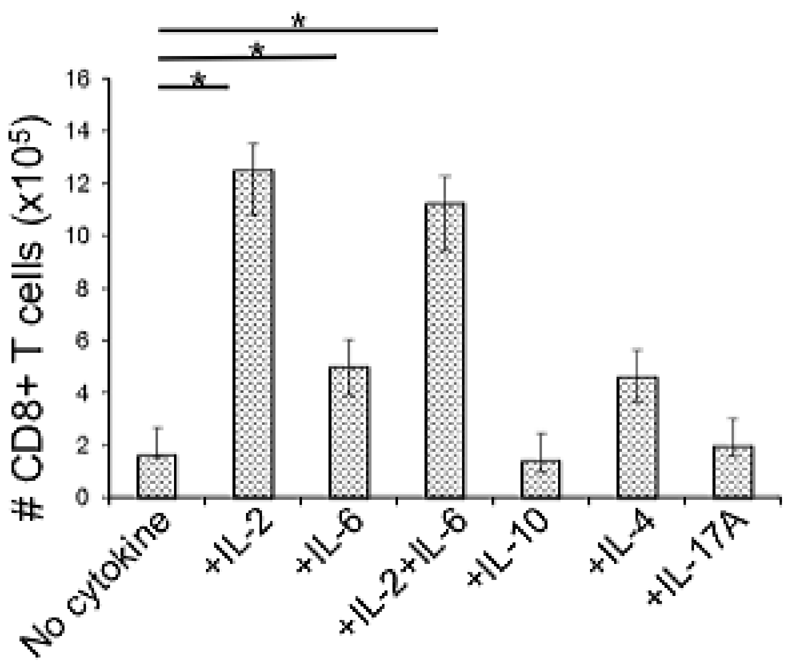

Supplemental Figure 1: The restorative effect of recombinant cytokines on aged CD8$^+$ T cells and the proliferative capacity of its effector memory subset in young adults. Number of CD8$^+$ T cells detected after 7 days of virus stimulation with or without recombinant cytokines. Human CD8$^+$ PBMCs (n=5/group0 from healthy young adults were sorted and stimulated with influenza H3N2 live virus for 7 days, and then total cell numbers were counted. *$p<0.0001$. Error bars represent standard error of the mean.
